# Breast Cancer Prevention Practices and Knowledge in Italian and Chinese Women in Italy: Clinical Checkups, Free NHS Screening Adherence, and Breast Self-Examination (BSE)

**DOI:** 10.1007/s13187-024-02463-4

**Published:** 2024-06-26

**Authors:** Luana Conte, Roberto Lupo, Alessia Lezzi, Serena Sciolti, Ivan Rubbi, Maicol Carvello, Antonino Calabrò, Stefano Botti, Annarita Fanizzi, Raffaella Massafra, Elsa Vitale, Giorgio De Nunzio

**Affiliations:** 1 Laboratory of Biomedical Physics and Environment, Department of Mathematics and Physics “E. De Giorgi”, University of Salento, Lecce, Italy; 2 Laboratory of Advanced Data Analysis for Medicine (ADAM) at the Laboratory of Interdisciplinary Research Applied to Medicine, University of Salento and Local Health Authority, Lecce, Italy; 3 “San Giuseppe da Copertino” Hospital, Local Health Authority, Copertino, Lecce Italy; 4 ANT Italia ONLUS Foundation (National Cancer Association), Lecce, Italy; 5 Lecce, Italy; 6 School of Nursing, University of Bologna, Faenza, Italy; 7 Community Hospital, Local Health Authority, Romagna, Italy; 8 “Nuovo Ospedale Degli Infermi” Hospital, Local Health Authority, Biella, Italy; 9 Haematology Unity, AUSL-IRCCS, Reggio Emilia, Italy; 10 Laboratorio di Biostatistica e Bioinformatica, IRCCS Istituto Tumori “Giovanni Paolo II”, Bari, Italy; 11 Scientific Directorate, IRCCS Istituto Tumori “Giovanni Paolo II”, Bari, Italy

**Keywords:** Breast cancer prevention, Screening adherence, Italian and Chinese women, Patient education, Public education, Professional education

## Abstract

Breast cancer remains a significant global concern, underscoring the critical need for early detection and prevention strategies. Primary and secondary preventive measures, such as routine screenings and behaviors like breast self-examination (BSE), play a crucial role in facilitating early diagnosis. While the National Health System (NHS) in Italy offers free regular screenings for women aged 50–69, there is a lack of clarity regarding the participation of both Italian and Chinese women residing in Italy in these screening programs. This study aims to bridge this knowledge gap by thoroughly assessing the involvement in regular clinical check-ups and the types of screening employed, the adherence to free screenings offered by the NHS, and the practice of BSE among women aged 50–69 of these two groups. Furthermore, it investigates their knowledge and perceptions regarding breast cancer and BSE. Results reveal disparities in breast cancer control practice between Italian and Chinese women in Italy: the former demonstrates higher adherence to clinical checkups (53% vs. 3%, *p* < 0.001), while both groups show low participation in free NHS screenings (70% vs. 4%, *p* < 0.001). Additionally, Chinese women reported significantly lower frequency of mammography (96% vs. 33%, *p* < 0.001) and ultrasound (69% vs. 16%, *p* < 0.001). The frequency of BSE also differed substantially, with 47% of Chinese women never performing BSE compared to 12% of Italian women (*p* < 0.001). This comprehensive exploration provides valuable insights, attitudes, and knowledge into the disparities and potential areas for improvement in breast cancer prevention, thus contributing to the overall well-being of these communities. The findings highlight the necessity for educational initiatives aimed at improving awareness and participation in screenings, particularly among the Chinese population. These initiatives could have profound implications for patient education by equipping women with the knowledge and skills necessary to engage in proactive health behaviors.

## Introduction

Breast cancer continues to be a significant global health concern, with a substantial impact on both diagnoses and mortality [1, 2]. Globally, it is the most frequently diagnosed cancer among women and ranks as the fifth leading cause of cancer-related deaths, resulting in approximately 2.3 million new cases and 685,000 deaths each year [3]. Breast cancer accounts for a significant portion of cancer diagnoses in women, representing one out of every four cases and causing one in six cancer-related deaths. Statistically, 1 in 18 women worldwide will experience a breast cancer diagnosis in their lifetime [4]. In Italy, the burden of breast cancer remains significant, with an estimated 55,700 new diagnoses and 12,500 deaths expected in 2022 [5]. Despite heightened awareness, public health efforts, and new technologies, breast cancer continues to be a major cause of mortality in Italy, with it being reported as the leading cause of death [5–10]. Shifting our focus to China, breast cancer poses a significant health challenge for its population. Data from GLOBOCAN 2020 reveals that it is the most common cancer among Chinese women, with 416,371 new cases and 117,174 deaths reported in 2020. These numbers account for a substantial portion of the global burden, with 18% of new cases and 17% of global deaths attributed to breast cancer [11]. Moreover, the incidence and mortality rates for breast cancer in Chinese women are increasing, and diagnoses often occur at a relatively young age [12, 13]. This situation emphasizes the necessity of establishing standardized breast cancer screening programs for Chinese women, commencing at an earlier age. However, when considering Chinese women residing in Italy, our knowledge about breast cancer incidence, diagnosis, and treatment outcomes in this specific group remains limited. This knowledge gap underscores the need for targeted research to better understand and address the unique challenges and needs of Chinese women in Italy concerning breast cancer. Educational programs are essential to address these challenges and enhance breast cancer awareness and prevention strategies.

In Italy, in accordance with preventive recommendations, women aged 50–69 have access to free biennial mammography screenings. Despite the potential benefits, not all eligible women take advantage of this service, resulting in pronounced regional variations in screening participation, breast cancer incidence, and survival outcomes [14, 15]. One contributing factor to this inequality could be a lack of knowledge about cancer risk factors, symptoms, and preventive strategies. A European survey has highlighted insufficient awareness in Italy about routine screening tests, underscoring the need for enhanced education and awareness initiatives [16].

Another highly recommended preventive measure is breast self-examination (BSE), a practice in which women routinely inspect their own breasts for any unusual changes and promptly report any findings to their healthcare provider. While BSE alone may not be as comprehensive as advanced diagnostic tools such as mammography and breast ultrasounds, it can significantly reduce the risk of late-stage breast cancer detection when performed consistently and accurately. Additionally, BSE is a practical and cost-effective method, making it accessible to a broader population. Women are encouraged to initiate BSE as early as possible [17], and education about the advantages of BSE is vital, as it represents the simplest and most direct approach to detecting breast cancer at an early, more treatable stage [17, 18].

In light of the persistent disparities in breast cancer screening uptake, there is a compelling need for interventions that are culturally and linguistically tailored to the specific demographics they aim to serve. Educational programs play a pivotal role in enhancing breast cancer awareness and screening participation, particularly among underserved populations. Previous studies have demonstrated the effectiveness of culturally tailored educational interventions in increasing knowledge and engagement in preventive behaviors. For instance, community-based educational initiatives have significantly improved BSE practices and mammography screening rates among various demographic groups [19]. In Shanghai, randomized trials have shown that educational efforts can lead to substantial improvements in BSE practices and early cancer detection [20]. Additionally, European women have exhibited increased awareness of cancer risks and screening benefits following targeted online interventions [16].

In Italy, educational programs have been shown to be effective in improving breast cancer screening practices among women. A study by Conte et al. highlighted the role of educational tools and technological interventions in promoting BSE and population screening [17]. Moreover, interventions designed to address barriers such as lack of awareness, cultural beliefs, and accessibility to healthcare services have been crucial in increasing screening participation among various ethnic groups [21].

These findings underscore the necessity for implementing culturally and linguistically appropriate educational programs to address the specific needs of Chinese women residing in Italy, thereby bridging the knowledge gap and promoting early breast cancer detection [22]. Educational initiatives should also incorporate strategies to engage community leaders and healthcare providers in spreading awareness and encouraging participation in screening programs [15]. Tailored interventions that consider the unique cultural and linguistic context of Chinese women can significantly enhance the effectiveness of these programs, leading to better health outcomes and reduced disparities in breast cancer prevention and control. The aim of this study is to investigate and compare the attitudes, behaviors, and knowledge of Chinese and Italian women aged 50–69 living in Italy regarding breast cancer control practices, participation in free screening programs offered by the NHS, and the practice of BSE. Additionally, the study aims to assess their knowledge and perceptions related to breast cancer and BSE. This research seeks to identify disparities between these two populations in order to enhance breast cancer prevention and early detection strategies in both groups, with a particular focus on educational implications. By bridging this knowledge gap, this study will empower healthcare providers, policymakers, and the community itself to make informed decisions and work collectively toward improved breast health outcomes for Italian and Chinese women in Italy.

## Methods

### Study Design

From April 2023 to October 2023, we conducted a survey targeting Chinese women residing in Italy, following a methodology outlined in our previous study [17]. Indeed, the same questionnaire, previously administered to Italian participants, was used to collect responses from the Chinese participants. All details about the survey instruments were found in [17]. Briefly, the questionnaire was digitalized using a predefined form on the Google Drive platform, and the survey was disseminated electronically. Various Facebook groups and Instagram pages were approached for circulating the digital questionnaire. The sampling technique employed was the (virtual) snowball method, which continued until we reached data saturation.

For this study, we specifically focused on the comparison among Italian and Chinese women in the 50–69 age group, as they are the primary recipients of Italian free breast cancer screenings. Among the Chinese participants, 230 women fell into the 50–69 age range. Among the Italian participants included in the previous study [17], 338 women were in this age range, and all of them were included in this study.

### Survey Instruments

The survey instrument was employed to compare Italian and Chinese women. Notably, four sections — that assessed both attitudes and knowledge regarding breast cancer prevention practices — were adapted from the original survey instrument [17]:

1. *Socio-demographic data* (8 items): This section encompassed inquiries about age, geographical area of residence, marital status, level of education, employment status, previous breast diseases, the presence of relatives with breast cancer, and a family history of cancer.
2. *Clinical checkups and free screening adherence* (7 items): This section delved into the clinical checkups conducted, the type of screening undergone, and adherence to free screenings offered by the NHS.
3. *Knowledge of tumors* (2 items): This section sought to understand their expressed knowledge regarding Breast Cancer and BSE.
4. *Breast self-examination* (3 items): This section concentrated on the practice and perception of BSE.

Through these sections, we conducted a comprehensive analysis to explore the differences and similarities in breast cancer prevention and early detection practices among Italian and Chinese women aged 50–69.

### Ethical Considerations

The ethical aspects of the study were clearly explained in the questionnaire introduction. The questionnaire design followed the guidelines established by the Italian Data Protection Authority (DPA). It was emphasized that participation was entirely voluntary, and participants had the option to withdraw from the study at any point. Individuals who expressed their interest in participating were provided with an informed consent form that reiterated the voluntary nature of their involvement and guaranteed the confidentiality and anonymity of the data collected. Additionally, to enhance the protection of participants' privacy, all questionnaire responses were made anonymous.

### Statistical Analysis

The study involved two distinct groups: Group A representing the Chinese population (*n* = 230), and Group B representing the Italian population (*n* = 338). Descriptive statistical analysis was applied to the data collected from the questionnaire responses. Continuous variables were expressed as the mean and standard deviation (SD), while categorical variables were presented as frequencies and percentages.

To identify differences between the two groups, the Mann–Whitney *U*-test was employed. Furthermore, within each group, we examined potential factors influencing clinical check-up participation, adherence to free screenings, and breast self-examination (BSE) using Fisher’s exact tests.

To delve deeper into the influencing factors identified by the Fisher’s exact tests, we conducted multiple linear regression analysis. In this analysis, the influencing factors were treated as independent variables, while clinical check-up participation, adherence to free screenings, and BSE were each examined as separate dependent variables. This approach allowed for a more detailed examination of how these influencing factors may independently impact each of these outcomes.

Statistical significance was determined at a threshold of *p* < 0.05. All statistical computations, encompassing both qualitative and quantitative data analyses, were conducted using MATLAB software.

## Results

### Baseline Characteristics and Questionnaire Items

The results revealed significant disparities in both attitudes and knowledge regarding breast cancer prevention practices between Italian and Chinese women. Italian women demonstrated higher adherence to clinical checkups and screenings, while Chinese women showed lower levels of knowledge and less frequent engagement in BSE.

Socio-demographics and questionnaire items for Chinese and Italian groups are separately reported in Table 1 whereas statistical differences are reported in Table 2.

**Table 1:** Baseline characteristics and the questionnaire items of all respondents aged 50–69

	Group A: Chinese population aged 50–69 (*n* = 230) *N* (%)	Group B: Italian population aged 50–69 (*n* = 338) *N* (%)
*Socio-demographics*		
Age (y)		
50–59 60–69	168 (73) 62 (27)	249 (74) 89 (26)
Geographic area		
North Center South/Islands	607 (26) 512 (22) 1256 (53)	100 (30) 104 (31) 134 (40)
Marital status		
Married Divorced Maiden Separate Widow	147 (64) 26 (11) 6 (3) 31 (13) 20 (9)	225 (67) 41 (12) 35 (10) 18 (5) 19 (6)
Education level		
Degree High school graduation Junior high school diploma Primary school None	5 (2) 12 (5) 175 (76) 33 (14) 5 (2)	101 (30) 154 (46) 73 (22) 8 (2) 2 (2)
Employment status		
Public Administration Services/Tertiary Student Retired Freelancer Unemployed Other	3 (1) 17 (7) 3 (1) 113 (49) 77 (34) 16 (7)	121 (36) 96 (28) 0 46 (14) 75 (22) 0
Previous breast issues		
Breast cancer Presence of a palpable lump Nipple discharge Breast pain (mastodynia) Other I have never had any issues	13 (6) 21 (9) 10 (4) 14 (6) 53 (23) 119 (52)	60 (18) 54 (16) 11 (3) 32 (9) 3 (1) 178 (53)
Relatives with tumors		
No Yes	160 (70) 70 (30)	195 (57) 143 (42)
Is there a family history of cancer?		
No Yes	160 (70) 70 (30)	195 (58) 143 (42)
*Clinical checkups and screening adherence*		
Have you ever done clinical checkups for early detection of breast cancer?		
Never Rarely Occasionally Often Always	107 (47) 103 (45) 0 14 (6) 6 (3)	20 (6) 17 (5) 45 (13) 78 (23) 178 (53)
If yes, please indicate the frequency		
I have never had a screening exam Every 2 years Every year Every 6 months Every month	105 (46) 75 (33) 39 (17) 8 (3) 3 (1)	17 (5) 140 (41) 164 (49) 15 (4) 2 (1)
Have you ever taken advantage of the free screening offered by the region?		
No Yes I don’t know them	220 (96) 10 (4) 0	97 (29) 235 (70) 6 (2)
*Type of screening performed*		
Have you ever had a mammogram?		
No Yes Missing	155 (67) 75 (33) 0	6 (2) 323 (96) 9 (2)
Have you ever performed an ultrasound?		
No Yes Missing	192 (83) 38 (16) 0	51 (15) 235 (69) 52 (15)
Have you ever performed an magnetic resonance imaging (MRI)?		
No Yes Missing	209 (91) 21 (9) 0	169 (50) 25 (7) 144 (43)
Have you ever had a biopsy?		
No Yes Missing	170 (74) 73 (32) 0	151 (45) 56 (17) 131 (39)
*Knowledge of tumors*		
Have you ever heard about breast cancer?		
No Yes	10 (4) 220 (96)	7 (2) 331 (98)
Do you think you are well informed about breast cancer prevention?		
A lot Sufficient Little Not at all	8 (3) 23 (10) 189 (82) 10 (4)	39 (12) 238 (70) 55 (16) 6 (2)
*Breast self-palpation (BSE)*		
Do you know and are well informed about BSE?		
No Yes	23 (10) 207 (90)	15 (4) 323 (96)
How often do you perform breast self-examination?		
Never Rarely Occasionally Often Always	107 (47) 103 (45) 14 (6) 6 (3)	42 (12) 177 (52) 107 (32) 12 (4)
Do you consider BSE useful for breast cancer prevention?		
No Yes	17 (7) 213 (93)	41 (12) 297 (88)

**Table 2:** Differences in screening participation among adult females aged 50–69 in Chinese subjects (Group A, *n* = 230) and Italian subjects (Group B, *n* = 338). Statistical significance was determined by the Mann–Whitney test (*p < 0.05; **p < 0.01; ***p < 0.001)

	*Z*-value	Rank sum	*p*-value
*Socio-demographics*			
Age Geographical area Marital status Education level Occupational status Previous breast cancer diagnosis Relatives with tumors	− 0.16 3.76 − 1.18 2.71 − 9.99 − 23.81 2.86	95.918 102.896 9.42e + 04 101,058 7.74e + 04 100,864 100,776	0.86 < 0.001*** 0.23 0.006** < 0.001*** < 0.001*** 0.004**
*Clinical checkups and free screening adherence*			
Underwent clinical checkups Frequency of clinical checkups Adherence to screenings offered by the NHS	14.6044 10.8385 15.6765	123,133 1.15e + 05 122,156	< 0.001*** < 0.001*** < 0.001***
*Type of screenings performed*			
Mammography Ultrasound Magnetic resonance imaging (MRI) Biopsy	12.1534 12.4001 − 0.74 − 3.67	112,857 116,764 95,487 91,109	< 0.001*** < 0.001*** 0.45 < 0.001***
*Knowledge of tumors*			
Heard of breast cancer Being informed about breast cancer	1.56 − 15.73	97,046 6.87e + 04	0.11 < 0.001***
*Breast self-examination (BSE)*			
Being informed about BSE Frequency of BSE Consider BSE useful	2.60 10.06 − 1.82	98,323 1.14e + 05 94,319	< 0.001*** < 0.001*** 0.06

Regarding baseline characteristics, most women in both groups came from the northern regions, with 53% of the Chinese group and 40% of the Italian group residing there. However, significant differences in geographical area among the two groups were observed (*p* < 0.001). Differences in marital status were minor, with a slightly higher percentage of married Italian participants. A distinct difference (*p* = 0.006) was observed in education level. Most Chinese women had a junior high school diploma, while Italian women were primarily high school graduates (46%) or degree holders (30%). Differences were also evident in employment status (*p* < 0.001). The majority of Chinese women were retired (49%), while Italian women were still working, especially in public administration (36%). Regarding previous breast issues and family history of cancer, differences were found for both groups (*p* < 0.001 and *p* = 0.004, respectively), with the majority reporting never having had breast issues (52% and 53% for Chinese and Italian groups, respectively) or a family history of cancer (70% vs. 58%, respectively). In addition, among the two groups, 18% of Italians reported a history of breast cancer, compared to 6% of Chinese.

**Fig. 1:**
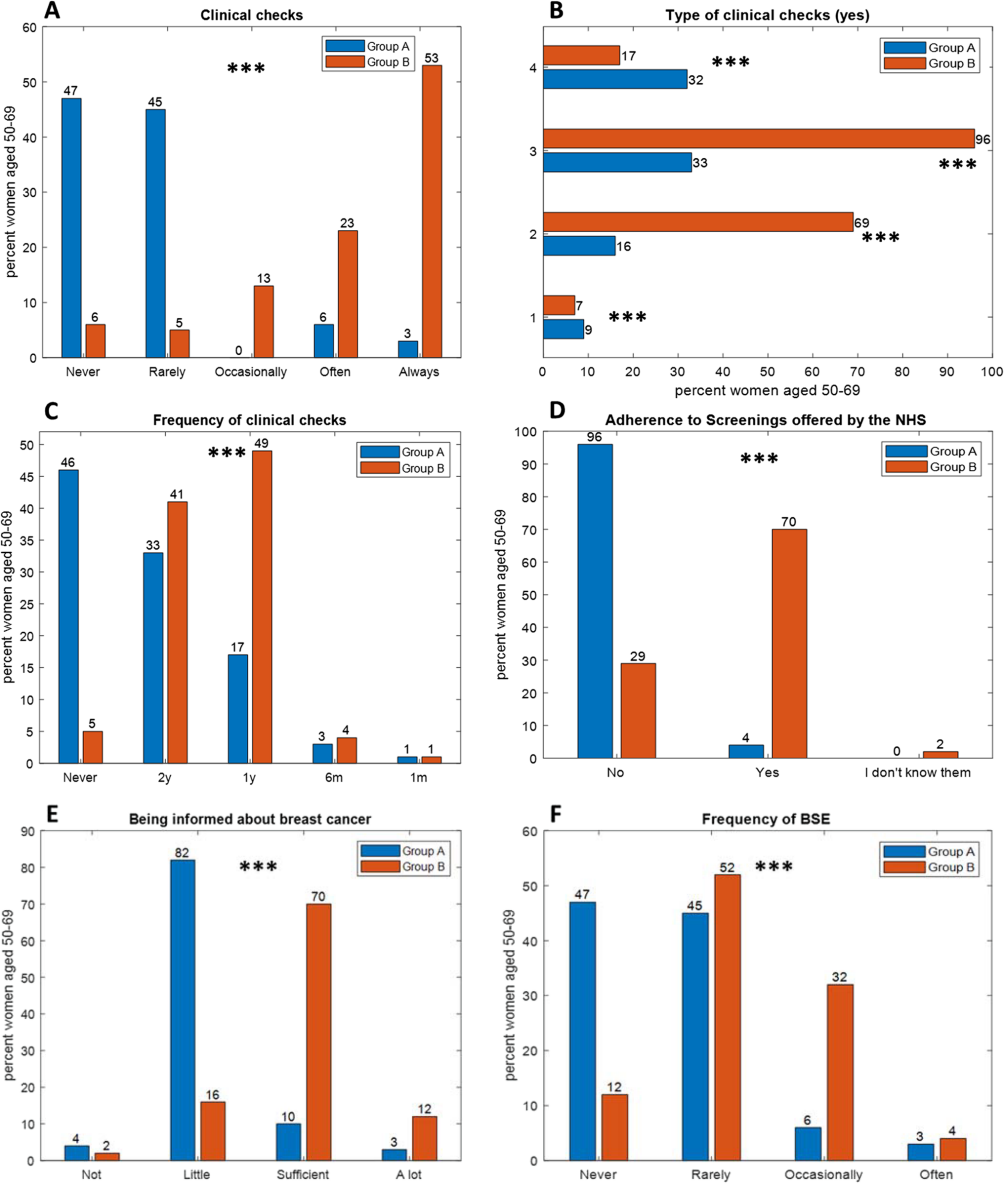
Participants were divided into Group A (Chinese population) in Blue and Group B (Italian population) in Red. Panel **A:** Clinical breast cancer controls, Panel **B:** type of clinical checkups (1, magnetic resonance imaging (MRI); 2, ultrasound; 3, mammogram; 4, biopsy). Only “Yes” responses are shown. Panel **C:** Frequencies of clinical checkups (y, years; m, months). Panel **D:** Adherence to the free screening program offered by the National Health Service (NHS). Panel **E:** Being informed about breast cancer. Panel **F:** Frequencies of performing Brest self-examination (BSE). A *p* value < 0.05 was considered statistically significant (*p < 0.05; **p < 0.01; ***p < 0.001)

Clinical checkups for early breast cancer detection showed substantial differences between the two groups (Fig. 1, Panel A). A significant portion of Chinese women does not engage in clinical checkups, with 47% of them reporting they have “Never” done these checks, while another 45% reported doing them “Rarely.” In contrast, a majority of Italian women (53%) reported they engage in clinical checkups “Always,” (*p* < 0.001). Also for the frequency of screening exams (Fig. 1, Panel C), Chinese and Italian women displayed different patterns. The majority of Chinese women (46%) claimed they have “Never” had a screening exam, with only 6% reporting “Always.” In contrast, Italian women reported more consistent screening participation, with 76% reporting they performed screenings “Often” or “Always,” (*p* < 0.001). Notable differences emerged also in the types of screenings performed (*p* < 0.001) among the two groups (Fig. 1, Panel B).

For the utilization of free regional screenings, both groups showed a low utilization of free regional screenings (Fig. 1, Panel D). A substantial percentage of Chinese women (96%) and Italian women (71%) stated they have “Never” taken advantage of these free screenings (*p* < 0.001). These findings highlight variations in screening behaviors and awareness between the two groups.

Continuing the analysis, when examining the level of knowledge about breast cancer (Fig. 1, Panel E), although both groups had heard about breast cancer, it was found that the majority of Chinese women (82%) considered themselves “Little” informed, while the majority of Italian women (70%) believed they were “Sufficient” informed about breast cancer prevention (*p* < 0.001). These findings underscore differences in perceived knowledge and awareness regarding breast cancer between the Chinese and Italian participants.

The perceived information about BSE also differed significantly between the Chinese and Italian participants (*p* < 0.001). When it comes to the frequency of performing BSE (Fig. 1, Panel F), the two groups displayed varying behaviors: a significant portion of Chinese women reported that they “Never” (45%) perform BSE. In contrast, the majority of Italian women (84%) reported that they perform it “Occasionally” or “Often.”

Regarding the perception of the usefulness of BSE for breast cancer prevention, both groups overwhelmingly agreed on its significance. In the Chinese group, 93% found BSE useful, and in the Italian group, 88% held the same opinion. While there were no substantial differences in this aspect, the shared acknowledgment of the value of BSE for breast cancer prevention is a noteworthy finding.

We further investigate factors influencing clinical checkup adherence, participation in free screenings offered by the NHS, and BSE within Group A and Group B independently. We explored a range of variables, including socio-demographic factors, knowledge about tumor and BSE, and the attitudes of performing BSE. The results are shown in Table 3.

**Table 3:** Factors influencing clinical checkups, regional free screening participation, and breast-self palpation (BSE) among two populations independently: Group A (Chinese population aged 50–69) and Group B (Italian population aged 50–69). Odds ratios (OR), *p*-values, and 95% confidence intervals (CI) are reported for each predictor category. Fisher’s exact tests were employed for statistical analysis. Significance levels: *p < 0.05, **p < 0.01, ***p < 0.001

Clinical checkups	Group A — Chinese population aged 50–69 (*n* = 230)			Group B — Italian population aged 50–69 (*n* = 338)		
	OR	*p*-value	CI 95%	OR	*p*-value	CI 95%
*Socio-demographics*						
Geographical area Education level Occupational status Previous breast cancer diagnosis Relatives with tumors Previous breast pathologies	0.68 146 0.05 4.75 5.81 0.005	0.72 < 0.001*** < 0.001*** 0.10 0.01** < 0.001***	0.18–2.50 14.11–1.51e + 03 0.01–0.27 0.89–25.07 1.45–23.19 0.001–0.01	0.39 0 1.51 1.17 0.96 0.90	0.001*** 1 0.10 0.64 0.90 1	0.22–0.70 – 0.93–2.53 0.64–2.12 0.60–1.54 0.36–2.24
*Knowledge of tumors*						
Heard of breast cancer Being informed about breast cancer	23.77 7.46	< 0.001*** 0.005**	5.29–106.87 2.02–27.52	5.94 2.84	0.02* 0.002**	1.13–31.15 1.40–4.39
*Breast self-palpation (BSE)*						
Being informed about BSE Frequency of BSE Consider BSE useful	7.05 0.11 49	0.01** 0.02* < 0.01***	1.82–27.19 0.01–0.95 11–218	4.94 2.33 3.08	0.003** 0.01* < 0.001***	1.64–14.86 1.21–4.49 1.58–6.003
Adherence to screening offered by the NHS	Group A — Chinese population aged 50–69 (*n* = 230)			Group B — Italian population aged 50–69 (*n* = 338)		
	OR	*p*–value	CI 95%	OR	*p*–value	CI 95%
*Socio-demographics*						
Geographical area Education level Occupational status Previous breast cancer diagnosis Relatives with tumors Previous breast pathologies	0.78 0 1.25 0.09 0.94 0.43	0.39 0.06 0.52 0.007** 0.88 0.29	0.44–1.37 – 0.66–2.38 0.01–0.72 0.53–1.66 0.12–1.56	0.78 0 1.03 1.16 0.56 2.57	0.80 1 1 0.76 0.35 0.002**	0.27–2.21 - 0.40–2.67 0.37–3.63 0.21–1.51 1.40–4.72
*Knowledge of tumors*						
Heard of breast cancer Being informed about breast cancer	0 0.19	0.002** < 0.001***	– 0.07–0.52	6.95 418	0.05* 0.003**	1.26–38.35 1.65–10.60
*Breast self-palpation (BSE)*						
Being informed about BSE Frequency of BSE Consider BSE useful	0.30 673.33 0.06 0.11	0.02* < 0.001*** < 0.001***	0.10–0.84 164–2.76e + 03 0.008–0.51	4.50 2.35 3.46	0.05* 0.08 0.02*	1.15–17.46 0.86–7.37 1.25–9.59
Breast self-palpation (BSE)	Group A — Chinese population aged 50–69 (*n* = 230)			Group B — Italian population aged 50–69 (*n* = 338)		
	OR	*p*–value	CI 95%	OR	*p*–value	CI 95%
*Socio-demographics*						
Geographical area Education level Occupational status Previous breast cancer diagnosis Relatives with tumors Previous breast pathologies	0.69 6.47 0.37 6.90 1.24 0.20	0.48 0.07 0.05* 0.004** 0.63 < 0.001***	0.28–1.69 1.02–40.97 0.15–0.92 2.04–23.33 0.50–3.09 0.08–0.50	0.35 23 1.12 0.70 0.67 0.97	0.24 0.08 1 1 0.59 1	0.07–0.59 1.36–387.02 0.37–3.35 0.15–3.19 0.22–2.00 0.34–2.74
*Knowledge of tumors*						
Heard of breast cancer Being informed about breast cancer	29.75 10.79	< 0.001*** < 0.001***	7.01–126.20 4.19–27.75	19.93 7.81	0.002** < 0.001***	4.00–99.15 2.66–22.89
*Breast self-palpation (BSE)*						
Frequency of BSE Considering BSE useful	0.87 21.97	0.82 < 0.001**	0.36–2.07 7.19–67.15	7.2 5.48	< 0.001*** 0.004**	2.46–21.06 1.84–16.33

Socio-demographic characteristics revealed intriguing differences and similarities between these two groups, shedding light on the determinants of clinical checkup behavior. For Group A, socio-demographics played a significant role in clinical checkup adherence. Educational level (*p* < 0.001), occupational status (*p* < 0.001), relatives with tumors (*p* = 0.01), and previous breast pathology (*p* < 0.001) emerged as key factors influencing clinical checkups. Among these, educational level appeared to be particularly impactful, indicating a strong association between higher education and a greater likelihood of participating in clinical checkups. Moreover, occupational status and a history of breast pathology were also strongly connected with clinical co-checkup control adherence among Chinese women. In contrast, for the Italian population, the sole socio-demographic factor significantly affecting clinical checkups was geographical area, with an odds ratio (OR) of 0.39 (*p* < 0.001). This suggests that specific regions play a more prominent role in influencing clinical checkup adherence among Italian women, resulting in a 61% lower likelihood of participation for Chinese women residing in these areas.

Moving on to the adherence to screening offered by the NHS, previous breast cancer diagnosis emerged as a strong influencing factor (*p* < 0.007) for the Chinese population. Conversely, previous breast pathologies exhibited a significant association with screening participation in Group B (*p* < 0.002). These pathologies include also cancer diagnoses, indicating that Chinese women are more inclined to participate in screening when there is a cancer assessment involved.

When considering BSE, previous breast cancer (*p* = 0.004) and previous breast pathologies (*p* < 0.001) were strongly associated with BSE practice for the Chinese group, along with occupational status. These results suggest that a history of breast cancer and other pathologies, combined with specific occupational roles, play pivotal roles in motivating Chinese women to practice BSE. Furthermore, higher education was found to positively influence the likelihood of self-palpation. In contrast, for the Italian group, no socio-demographic factors appeared to influence BSE attitudes, highlighting a contrast between the two populations in terms of BSE practices.

While socio-demographic factors demonstrated varying impacts, both populations underscored the importance of knowledge and awareness in influencing clinical checkup behavior. Those who had heard about and were informed about breast cancer displayed a higher likelihood of undergoing clinical checkups. Knowledge about tumors was found to be influential in screening participation and BSE practices for both groups. This highlights the significance of public awareness campaigns in encouraging screenings and promoting the practice of BSE.

Similarly, being informed about BSE, considering it useful, and the frequency of BSE showed a more significant impact for both groups, emphasizing the role of self-examination in promoting screening.

To further analyze the influencing factors identified as predictor variables for clinical checkup adherence and participation in screenings offered by NHS and BSE practice, a multiple linear regression analysis was performed (Table 4).

**Table 4:** Multiple linear regression analysis was performed with the predictor variables and the clinical checkups, adherence to free screening offered by the NHS, and breast self-palpation (BSE) as dependent variables. A *p*-value < 0.05 was considered statistically significant (*p < 0.05; **p < 0.01; ***p < 0.001)

Independent predictors	Dependent variable	Group A — Chinese population aged 50–69 (*n* = 230)				Group B — Italian population aged 50–69 (*n* = 338)			
		*R*^2^	*R*^2^ adj	*F*-statistic	*p*-value	*R*^2^	*R*^2^ adj	*F*-statistic	*p*-value
*Socio-demographics*									
Geographical area Education level Occupational status Previous breast cancer diagnosis Relatives with tumors	Clinical checkups	0.87	0.86	167	< 0.001***	0.05	0.02	2.14	0.02*
*Knowledge of tumors*									
Heard of breast cancer Being informed about breast cancer	Adherence to Screenings offered by the NHS	0.46	0.44	21.2	< 0.001***	0.10	0.08	4.34	< 0.001***
*Breast Self-Palpation (BSE)*									
Being informed about BSE Frequency of BSE Considering BSE useful	Breast Self-Palpation (BSE)	0.27	0.24	9.04	< 0.001***	0.13	0.10	5.47	< 0.001***

For Chinese women, the model for clinical checkup adherence demonstrated a strong fit, with an impressive 87% of the variability explained by the predictor variables. Additionally, the *F*-statistic is substantial (*F*-statistic = 167, *p* < 0.001), reinforcing the model’s overall significance and reliability. In contrast, the model for Italian women in terms of clinical checkup adherence presents a different scenario. Only about 5% of the variability in clinical control adherence can be attributed to the predictor variables. While the *p*-value is statistically significant (*p* < 0.005), indicating that the model does provide some valuable insights, it is crucial to note that the significance level is significantly weaker than in the Chinese group. This suggests that the predictor variables have a weaker collective influence on clinical checkup adherence among Italian women, and other unaccounted factors may play a more prominent role in their clinical control decision-making.

Turning to the analysis of adherence to NHS-offered screenings, the Chinese group’s model demonstrates a relatively strong fit, explaining approximately 46% of the variability in screening adherence (*p* < 0.001). This reflects the significance of the predictor variables in influencing the decision to participate in these screenings. Conversely, the model for Italian women in terms of adherence to NHS-offered screenings explains only around 10.6% of the variability, which is significantly lower than the Chinese group. Although the *p*-value is statistically significant (*p* < 0.001), this relatively weaker explanatory power suggests that other unaccounted factors play a more substantial role in shaping Italian women’s decisions regarding NHS screenings.

Lastly, analysis of BSE practice among Chinese women reveals a model with moderate explanatory power. Approximately 27% of the variability in BSE could be explained by the predictor variables (*p* < 0.001). In contrast, for the Italian group, the model provides a lower level of explanatory power for BSE practice, explaining about 13.1% of the variability (*p* < 0.001). The difference in explanatory power between the two groups suggests that the predictor variables have a stronger collective influence on BSE practices among Chinese women compared to Italian women. This discrepancy may be attributed to cultural and contextual factors influencing health behaviors and awareness.

## Discussion

The study aimed to compare the attitudes and knowledge of Chinese and Italian women, particularly those aged 50–69, toward clinical breast cancer controls and adherence to free screening programs provided by the Italian NHS. Additionally, it examined women’s knowledge and awareness about cancer and BSE, as well as the frequency of BSE and the emotional approach to it.

The findings pertaining to clinical checkup adherence are of great significance. Chinese women exhibited low engagement in clinical checkups, with a substantial proportion reporting infrequent or no engagement in these checks. In contrast, Italian women demonstrated a higher level of adherence to clinical checkups, with the majority reporting regular participation. This discrepancy might be attributed to factors such as a lack of awareness, challenges in accessing healthcare services, or cultural beliefs that influence their perceptions of breast health. Educational interventions targeting Chinese women could help bridge this gap. In addition, disparities in clinical exam frequency and type of exam performed also highlight differing approaches to breast health management between Chinese and Italian women. This variance could be attributed to Italy’s well-established healthcare infrastructure, along with a higher level of awareness and education regarding the importance of clinical checkups in breast cancer detection. The observed disparities underscore the importance of tailored interventions to enhance clinical checkup adherence among Chinese women. Healthcare providers and policymakers should concentrate on awareness campaigns and the removal of barriers to healthcare access.

Regarding the adherence to free regional screenings, both Chinese and Italian women indicated low utilization, indicating the need for improvement in promoting and providing access to these services. The substantial percentage of women who have never utilized these screenings underscores the necessity for targeted outreach programs and initiatives. Educating Chinese women about the significance of early detection and the availability of free screenings could be instrumental in increasing their participation in clinical checkups.

Continuing the analysis, the perceived knowledge about breast cancer and the emotional approach to BSE revealed further distinctions between the Chinese and Italian participants. These differences are noteworthy and can provide insights into breast health management practices: while it was common for both groups to have heard about breast cancer, significant variations existed in how informed the participants felt. The majority of Chinese women (82%) considered themselves “Little” informed about breast cancer, while the majority of Italian women (70%) believed they were “Sufficiently” informed. It is crucial to note that these differences in perceived knowledge and awareness can influence attitudes toward clinical checkups, screening, and prevention behaviors. When women perceive themselves as having limited knowledge about breast cancer, they may be less likely to engage in proactive health-seeking behaviors, such as clinical controls and screenings. In contrast, those who feel sufficiently informed are more likely to understand the significance of early detection, preventive measures, and the importance of regular clinical check-ups and screenings. As a result, interventions aimed at improving breast health behaviors should consider not only providing information but also boosting women’s confidence in their knowledge about breast cancer. Educational campaigns should be designed to address these knowledge gaps and empower women with the information needed to take proactive steps in their breast health management.

Distinct perceptions of information and awareness about BSE also emerged between the Chinese and Italian groups. Chinese women, in particular, appeared to perceive a relative lack of information about or awareness of the practice of BSE compared to their Italian counterparts. This discrepancy in perceived information levels can significantly impact the likelihood of women engaging in BSE practices, thereby affecting early detection of breast abnormalities. Indeed, a substantial portion of Chinese women (45%) reported that they “Never” perform BSE. In contrast, the majority of Italian women (84%) reported that they perform self-examinations “Occasionally” or “Often.” This finding raises concerns, as BSE can serve as a valuable tool for the early detection of breast irregularities and potentially life-saving diagnoses. These variations emphasize the need for targeted interventions to enhance awareness and knowledge of BSE, especially among the Chinese population. Educational programs should focus on teaching the proper techniques and importance of BSE to encourage its regular practice.

An additional analysis delved deeper into the factors influencing clinical breast cancer controls, participation in NHS screening programs, and BSE within two distinct groups. Socio-demographic characteristics played a significant role in clinical checkup adherence for Chinese women, with factors such as educational level, occupational status, family history of tumors, and previous breast pathology emerging as key determinants. This underscores the impact of educational attainment and personal health history on clinical checkup engagement in the Chinese population. Higher educational attainment is associated with a greater likelihood of participating in these clinical checks among Chinese women. This connection could be attributed to the fact that educated individuals often possess a better understanding of the importance of regular clinical checkups in breast cancer detection and are more likely to be aware of the significance of early diagnosis. In this context, educational campaigns and interventions tailored to different educational levels may be instrumental in increasing clinical checkup adherence, especially among less educated individuals. However, for Italian women, geographical area was the primary socio-demographic factor influencing clinical checkups, indicating the regional variation in adherence. In Italy, the healthcare system operates on a regional basis, and these regions may have differing approaches to promoting and providing healthcare services, including breast cancer screenings. This regional discrepancy suggests that certain areas within Italy might have more robust healthcare infrastructure, awareness campaigns, or accessibility to clinical checkup facilities, resulting in higher rates of adherence among Italian women residing in those regions. Conversely, regions with limited healthcare resources or lower awareness campaigns might see lower adherence rates. To address this variation, healthcare policies and interventions should be tailored to account for regional disparities. Emphasizing the importance of regular clinical checkups in breast cancer prevention and facilitating access to healthcare services should be part of these region-specific strategies. Additionally, promoting best practices from regions with higher adherence could benefit areas with lower rates and improve overall breast health management.

The distinction in the influencing factors for NHS screening participation between Chinese and Italian women underlines the importance of understanding how personal health experiences shape screening behavior. In the case of Chinese women, those with a previous breast cancer diagnosis exhibited a higher likelihood of participating in NHS screenings. This suggests that Chinese women with a history of breast cancer may be more vigilant about their health and proactive in seeking regular screenings as a preventive measure. On the other hand, the Italian group displayed a different pattern, with previous breast pathologies, including cancer diagnoses, significantly associated with screening participation. For Italian women, a history of breast abnormalities or cancer might serve as a motivator for engaging in regular screenings. This could be related to the heightened awareness of potential breast health issues and a proactive approach to monitoring health.

For BSE practices, a history of breast cancer, other pathologies, specific occupational roles, and higher education positively influenced Chinese women, while no socio-demographic factors appeared to affect Italian women’s BSE attitudes. These findings highlight the importance of tailored approaches to breast cancer screening promotion in each population and reinforce the central role of knowledge and awareness in shaping breast health behaviors.

Finally, The multiple linear regression analysis provided insights into how predictor variables explain women’s behaviors regarding clinical checkup adherence, NHS screening participation, and BSE practices. It revealed that for Chinese women, the predictor variables had a stronger explanatory power, indicating that they more effectively account for variations in these behaviors. In contrast, for Italian women, the predictor variables had weaker explanatory power, suggesting that other unexamined factors might play a more substantial role in influencing these behaviors. These findings emphasize the importance of recognizing the diverse factors that shape breast health management practices among these two populations and underscore the need for tailored approaches to enhance engagement in clinical checkups, NHS screenings, and BSE practices.

Based on the results, several future educational directions can be proposed: (i) Development of culturally tailored educational materials. The study reveals a considerable lack of awareness among Chinese women regarding the availability and importance of breast cancer screenings and BSE practices. Future educational initiatives should focus on developing and disseminating culturally tailored educational materials that are sensitive to linguistic needs and cultural nuances. This can involve translating existing materials into Mandarin and creating visual aids that reflect cultural relevancy, which could help in overcoming language and cultural barriers; (ii) community-based educational workshop. Organizing community-based workshops can be an effective way to reach out to these women, particularly in areas with high concentrations of Chinese residents. These workshops could cover topics such as the importance of regular screenings, how to perform BSE, and understanding common signs of breast cancer. Involving community leaders and healthcare providers who are culturally aligned with the audience can enhance the credibility and acceptance of the information; (iii) integration of education into healthcare services. Leveraging existing healthcare appointments as opportunities to educate women about breast cancer screening can ensure that all women, regardless of their background, receive the necessary information directly from healthcare professionals. This approach can be particularly effective in Italy’s public health system, where regular health checks can be complemented with educational sessions on the importance of screening and early detection strategies; (iv) use of digital platforms for education and outreach. Given the widespread use of digital platforms, creating online educational campaigns that include social media, videos, and interactive webinars can significantly expand the reach of educational messages. These platforms can be used to regularly update women on screening programs and changes in guidelines and provide reminders for scheduled screenings; (v) partnerships with local organizations and business. Collaborating with local organizations that work closely with the Chinese community, such as cultural associations and immigrant support groups, can help in effectively disseminating breast cancer prevention messages. Additionally, partnerships with local businesses that employ or cater to Chinese women can be a conduit for distributing educational materials and organizing informative sessions; (vi) long-term monitoring and feedback collection: To ensure the effectiveness of these educational programs, it is crucial to establish mechanisms for monitoring their impact and gathering feedback from the community. This could involve periodic surveys and focus groups to assess changes in awareness and behavior, and to refine educational strategies based on community feedback; (vii) educational training for healthcare providers: Training healthcare providers on cultural competence and communication skills can improve the quality of interactions with women from different cultural backgrounds. Educating providers about the specific challenges faced by the Chinese community in accessing cancer screening services will enable them to offer more personalized and effective support.

In conclusion, the differences in influencing factors between the two groups highlight the potential impact of cultural and contextual factors on health behavior. Understanding these differences can lead to more effective strategies for promoting breast health management and regular screenings in both populations. These educational directions are pivotal for future initiatives and could significantly impact public, patient, and professional education by fostering a more informed and proactive approach to breast health management among diverse populations.

## Limitations

It is important to consider certain limitations when interpreting the results of this study. Firstly, cultural and linguistic nuances, despite having a native speaker translate the questionnaire, might have been inadvertently overlooked or misinterpreted. Such nuances could have influenced how respondents comprehended and responded to specific questions.

Another limitation pertains to the potential lack of representation of a broader Chinese demographic. It is possible that individuals who are less integrated or less proficient in the dominant language may not have participated in the study. This limitation is closely associated with the mode of questionnaire administration through electronic means, which is likely more favored by younger women. The use of the snowball method is also a limitation of the study,

Furthermore, the choice of electronic questionnaire dissemination may have excluded individuals with limited computer literacy, potentially affecting the sample’s representativeness.

Lastly, it is important to acknowledge the possibility of non-response bias. Some respondents may have chosen not to participate in the survey due to cultural or personal reasons, which could influence the generalizability of the findings. Information bias might also have been introduced due to respondents’ reluctance to declare a lack of knowledge on the topic, affecting the accuracy of their responses.

## Conclusions

Addressing both attitudes and knowledge is crucial for improving breast cancer prevention practices among Chinese and Italian women in Italy. Our study highlights significant disparities in breast cancer control adherence, knowledge, and screening practices between Chinese and Italian women aged 50–69. Chinese women demonstrate lower engagement in clinical checkups and breast self-examination (BSE) compared to their Italian counterparts. Additionally, adherence to free screening programs offered by the Italian National Health Service (NHS) is notably low for both groups. While socio-demographic factors, such as educational level, occupational status, and family history, appear to influence Chinese women's clinical checkup adherence, regional variations significantly impact the Italian group. To bridge the gap in breast health management, particularly among Chinese women, comprehensive information campaigns and targeted educational initiatives are imperative. The key objectives include raising awareness about breast cancer, promoting early detection, and emphasizing the value of regular clinical checkups and screenings. Implementing educational programs in academic institutions and leveraging technology can facilitate the dissemination of knowledge, particularly among younger women. Furthermore, involving healthcare professionals, such as nurses, in preventive healthcare and educational endeavors can enhance the effectiveness of these initiatives.

Our study has shed light on the intricate interplay of cultural, healthcare system, and awareness factors influencing breast health management among these two groups. To further address these disparities and encourage early breast cancer detection, future research should delve deeper into the specific cultural, educational, and accessibility barriers that underlie these differences. By comprehensively understanding these factors, we can develop more targeted and effective strategies to promote breast health management and reduce disparities among Chinese and Italian women in Italy. These future directions aim not only to improve breast cancer prevention and control practices but also to build a more inclusive healthcare environment that respects and addresses the diverse needs of Italy’s multicultural population. By focusing on these educational priorities, healthcare policymakers and community leaders can work toward reducing the disparities in breast cancer outcomes and ensuring that all women have the knowledge and resources needed to make informed decisions about their health.
